# Activation of SIRT1 Alleviates Ferroptosis in the Early Brain Injury after Subarachnoid Hemorrhage

**DOI:** 10.1155/2022/9069825

**Published:** 2022-07-09

**Authors:** Bin Yuan, Xu-Dong Zhao, Jun-Da Shen, Shu-Juan Chen, Han-Yu Huang, Xiao-Ming Zhou, Yan-Ling Han, Long-Jiang Zhou, Xiao-Jie Lu, Qi Wu

**Affiliations:** ^1^Department of Neurosurgery, The Affiliated Wuxi No. 2 Hospital of Nanjing Medical University, Wuxi, 214002 Jiangsu, China; ^2^Department of Neurosurgery, Jinling Hospital, Nanjing Medical University, Nanjing, 210002 Jiangsu, China; ^3^Department of Neurosurgery, Jinling Hospital, Nanjing University, School of Medicine, Nanjing, 210002 Jiangsu, China; ^4^Neuromedical Research Center, Jiangnan University, Wuxi, 214122 Jiangsu, China

## Abstract

Ferroptosis is a regulated cell death that characterizes the lethal lipid peroxidation and iron overload, which may contribute to early brain injury (EBI) pathogenesis after subarachnoid hemorrhage (SAH). Although Sirtuin 1 (SIRT1), a class III histone deacetylase, has been proved to have endogenous neuroprotective effects on the EBI following SAH, the role of SIRT1 in ferroptosis has not been studied. Hence, we designed the current study to determine the role of ferroptosis in the EBI and explore the correlation between SIRT1 and ferroptosis after SAH. The pathways of ferroptosis were examined after experimental SAH *in vivo* (prechiasmatic cistern injection mouse model) and in HT-22 cells stimulated by oxyhemoglobin (oxyHb) *in vitro*. Then, ferrostatin-1 (Fer-1) was used further to determine the role of ferroptosis in EBI. Finally, we explored the correlation between SIRT1 and ferroptosis via regulating the expression of SIRT1 by resveratrol (RSV) and selisistat (SEL). Our results showed that ferroptosis was involved in the pathogenesis of EBI after SAH through multiple pathways, including acyl-CoA synthetase long-chain family member 4 (ACSL4) activation, iron metabolism disturbance, and the downregulation of glutathione peroxidase 4 (GPX4) and ferroptosis suppressor protein 1 (FSP1). Inhibition of ferroptosis by Fer-1 significantly alleviated oxidative stress-mediated brain injury. SIRT1 activation could suppress SAH-induced ferroptosis by upregulating the expression of GPX4 and FSP1. Therefore, ferroptosis could be a potential therapeutic target for SAH, and SIRT1 activation is a promising method to inhibit ferroptosis.

## 1. Introduction

Subarachnoid hemorrhage (SAH) is a devastating cerebrovascular disease mainly caused by a ruptured intracranial aneurysm. Although SAH accounts for ~5% of all strokes, it has a high mortality (~35%); 30-50% of those who survived the initial bleeding still suffer long-term disability due to severe brain injury [1, 2]. The development of therapeutic techniques allows the ruptured aneurysms to be fully repaired by endovascular coiling or neurosurgical clipping. However, the residual blood and cleavage products still induce serious brain injury, leading to permanent neurological impairments. Although accumulating studies have reported that advanced technologies, such as nanotechnology and stem cell transplantation, can be used in treating neurological disorders, including SAH, these treatment methods did not significantly improve the prognosis of neuronal diseases [3–6]. SAH-induced brain injury includes early brain injury (EBI) and delayed brain injury. Increasing evidence indicates that EBI, referring to the entirety of brain injury within 72 h after SAH, is the most critical etiological factor of unfavorable clinical outcomes [7, 8]. The pathophysiological mechanisms of EBI are intricate and complex, involving oxidative stress, neuroinflammation, microcirculatory failure, excitotoxicity, mitochondrial dysfunction, and neuronal death [9]. Thereinto, oxidative stress responses resulting from the dysregulation of redox reactions substantially contribute to the process of EBI and are correlated to long-term outcomes [10].

Redox homeostasis regulates the normal physiological functions of cells. Excessive free radicals can exhaust the intrinsic antioxidant system, resulting in lipid peroxidation, DNA damage, and protein breakdown [11]. Ferroptosis is a new regulated cell death induced by excessive free radicals [12]. The essence of ferroptosis is lipid peroxidation accumulation, accompanied by iron overload [13]. The distinctive morphological features of ferroptosis cells are characterized as shrunken mitochondria with increased density of mitochondrial membrane [13]. The mechanisms that mediate ferroptosis are mainly divided into two categories: proferroptosis signaling pathways and antiferroptosis signaling pathways. The proferroptosis signaling pathways involve acyl-CoA synthetase long-chain family member 4- (ACSL4-) mediated abnormal fatty acid metabolism and iron metabolic dysfunction-induced Fenton reaction [14]. The previous study has demonstrated that transferrin receptor (TFR), divalent metal transporter 1 (DMT1), and ferroportin (FPN) regulate the intracellular iron level [15]. However, the specific role of these proteins in ferroptosis has not been clarified. Furthermore, the suppression of antiferroptosis pathways may be crucially responsible for the progression of ferroptosis, especially glutathione peroxidase 4- (GPX4-) and ferroptosis suppressor protein 1- (FSP1-) mediated antiferroptosis pathways [16]. GPX4 reduces the cytotoxic phospholipid hydroperoxide production to the corresponding phospholipid alcohol by converting glutathione (GSH) into oxidized glutathione (GSSG) [13]. GPX4 inhibition can cause the accumulation of lipid peroxides, which is considered the ferroptosis marker [17]. Ferroptosis suppressor protein 1 (FSP1) is recently identified as another antioxidant regulator involved in the process of ferroptosis. FSP1 catalyzes the regeneration of CoQ10 by NADPH, trapping lipid peroxides [18]. CoQ10H_2_ produced from the regeneration of CoQ10 is an effective lipophilic antioxidant involved in the suppression of ferroptosis [19]. Growing evidence has shown that ferroptosis is correlated with the process of several neurological diseases, and the inhibition of ferroptosis significantly alleviates these brain injuries [20–25]. However, the role of ferroptosis in SAH still needs to be thoroughly studied.

Sirtuin 1 (SIRT1) is a well-known epigenetic regulator that modulates gene transcription and impacts various biological functions, such as oxidative stress, inflammatory response, and mitochondrial biogenesis [26]. Numerous studies have shown that SIRT1 has a great antioxidative capacity and plays a neuroprotective role against EBI after SAH [11]. SIRT1 activation mitigates the oxidative stress-induced EBI by inhibiting p53- and nuclear factor-kappa B- (NF-*κ*B-) mediated oxidative pathways and upregulating the nuclear factor-erythroid 2-related factor 2 antioxidant pathway [27–29]. Although SIRT1 has been widely studied in EBI following SAH, there is still a lack of systemic study on SIRT1 and ferroptosis in EBI after SAH. Hence, to better understand how SIRT1 modulates ferroptosis in EBI following SAH, we used a mouse SAH model to assess the interplay of these molecular reactions *in vivo* and *in vitro*.

## 2. Methods and Materials

### 2.1. Chemicals and Reagents

Ferrostatin-1 (Fer-1, HY-100579), selisistat (SEL, HY-15452), and corn oil (HY-Y1888) were purchased from MedChemExpress (Shanghai, China). Resveratrol (RSV, R5010), dimethylsulfoxide (DMSO, V900090), and oxyhemoglobin (OxyHb, H2500) were purchased from Sigma-Aldrich (St. Louis, MO, USA).

### 2.2. Animal and SAH Model *In Vivo*

All animal experiments in this study were approved by the Ethics Committee of Wuxi No. 2 Hospital and were conducted in compliance with the ARRIVE guidelines (Animal Research: Reporting of *In Vivo* Experiments) and the National Institutes of Health Guidelines for the Care and Use of Laboratory Animals. Male C57BL/6 mice (8–10 weeks old) were brought from the Nanjing Biomedical Research Institute of Nanjing University.

SAH models *in vivo* were performed by prechiasmatic cistern injection of nonheparinized arterial blood [30]. After general anesthesia and scalp incision, a hole was drilled into the skull at 4.5 mm anterior to the bregma in the midline. 60 *μ*L nonheparinized arterial blood from a donor mouse was slowly injected into the prechiasmatic cistern. The hole was sealed with bone wax to prevent backflow or cerebrospinal fluid leakage. The sham controls were injected with 60 *μ*L of normal saline (NS).

### 2.3. Cell Culture and SAH Model *In Vitro*

HT-22 cells were purchased from Shanghai Zhongqiao Xinzhou Biotechnology Co., Ltd. and cultured in Dulbecco's modified Eagle's medium (DMEM, Thermo Fisher Scientific, Waltham, MA, USA) with 10% FBS and 1% penicillin-streptomycin at 37°C with 5% CO_2_.

OxyHb was dissolved in the DMEM, reaching the concentration of 20 *μ*M. Then, the cells were analyzed according to the experimental design after the cells were exposed to 20 *μ*M oxyHb.

### 2.4. Study Design and Drug Administration

#### 2.4.1. Experiment 1

The expression changes of ASCL4, TFR, DMT1, ferritin, FPN, XCT, GPX4, FSP1, COQ10B, and SIRT1 at different time points following SAH were determined *in vivo* and *in vitro*. Here, 150 mice were randomized into six groups: the sham, SAH 6 h, SAH 12 h, SAH 24 h, and SAH 72 h groups (*n* = 30/group). The HT-22 cells were randomized into five groups: the sham, oxyHb 6 h, oxyHb 12 h, oxyHb 24 h, and oxyHb 48 h groups.

#### 2.4.2. Experiment 2

The role of ferroptosis in EBI following SAH was evaluated. Forty-eight mice were randomized into four groups: the sham, SAH, SAH+vehicle(V), and SAH+Fer-1 groups (*n* = 12/group). The HT-22 cells were randomized into the sham, oxyHb, oxyHb+V, and oxyHb+Fer-1 groups.

For *in vivo* experiment, 3 *μ*L Fer-1(10 *μ*M) was slowly injected into the cerebral ventricle 2 h after SAH [31]. The vehicle group was injected with 3 *μ*L NS. The brain tissues were collected at 24 h after SAH. For *in vitro* experiment, Fer-1 at doses of 3 *μ*M, 6 *μ*M, 9 *μ*M, and 12 *μ*M was added to the DMEM with 20 *μ*M oxyHb. Then, the 20 *μ*M oxyHb with 9 *μ*M Fer-1 was performed in the follow-up experiments. The HT-22 cells were collected at 12 h after the oxyHb stimulation.

#### 2.4.3. Experiment 3

The expression of SIRT1 was observed after SAH. The role of SIRT1 activation in ferroptosis was also explored *in vivo* and *in vitro*. Forty-eight mice were randomized into four groups: the sham, SAH, SAH+V, and SAH+RSV groups (*n* = 12/group). The HT-22 cells were randomized into the sham, oxyHb, oxyHb+V, and oxyHb+RSV groups.

For the *in vivo* experiment, RSV (30 mg/kg) was administered daily by intraperitoneal injection for 3 days before surgery. The vehicle group was injected with corn oil. For the *in vitro* experiment, RSV at doses of 10 *μ*M, 30 *μ*M, 50 *μ*M, and100 *μ*M was added to the DMEM with 20 *μ*M oxyHb. Then, the 20 *μ*M oxyHb with 50 *μ*M RSV was performed in the follow-up experiments.

#### 2.4.4. Experiment 4

The role of SIRT1 inhibition in ferroptosis was determined *in vivo* and *in vitro*. Forty-eight mice were randomized into four groups: the sham, SAH, SAH+V, and SAH+SEL groups (*n* = 12/group). The HT-22 cells were randomized into the sham, oxyHb, oxyHb+V, and oxyHb+SEL groups.

For the *in vivo* experiment, SEL (10 mg/kg) was administered daily by intraperitoneal injection for 3 days before surgery [32]. The vehicle group was injected with corn oil. For the *in vitro* experiment, HT-22 cells were pretreated by SEL (10 *μ*M) for 2 h before the oxyHb stimulation.

### 2.5. Neurological Function Evaluation

Neurological function was evaluated at different time points (6, 12, 24, and 72 h) following SAH by two investigators blinded to the groups, as formerly reported [33]. An 18-point scoring system, ranging from 3 to 18, assessed the neurological deficits from six subtests. These tests comprise spontaneous activity, symmetry in all limbs' movements, forelimb extension, climbing, body proprioception, and response to vibrissae stimulation. Higher scores indicate better neurological function.

### 2.6. Brain Water Content Evaluation

Mice were killed by decapitation, and the brains were rapidly removed without perfusion. Filter paper gently blotted blood from the brain surface. The brains were immediately weighed as the wet weight (WW). Then, the dry weight (DW) was weighed after the brains were dried at 100°C for 72 h. The brain water content was calculated as follows: the percentage of brain water content (%) = [(WW–DW)/WW] × 100%.

### 2.7. Hematoxylin and Eosin (H&E) and Cresyl Violet (Nissl) Staining

H&E and Nissl staining were performed as formerly described [29, 34]. In brief, mice were transcardially perfused with 50 mL of cold NS and 4% paraformaldehyde at 24 h following SAH. Then, the brains were separated and fixed in 4% paraformaldehyde. After fixation, dehydration, and paraffin-embedding, the mouse brains were sliced into 4 *μ*m thickness sections. The sections were stained with H&E and Nissl according to standard procedures and then mounted with neutral resin (Beyotime, Shanghai, China). Finally, the sections were evaluated under a light microscope.

### 2.8. Immunofluorescence (IF) Staining

Immunofluorescence staining was performed as previously described [35]. Frozen brain sections (7 *μ*m) or cultured cells on coverslips were fixed in 4% paraformaldehyde for 10 min. Then, they were treated with Immunostaining Permeabilization Buffer with Triton X-100 (Beyotime, Shanghai, China) for 30 min and were blocked with Immunol Staining Blocking Buffer (Beyotime, Shanghai, China) for 60 min. These samples were incubated with specific primary antibodies for ACSL4 (Affinity, DF12141, 1 : 200), FTH (Affinity, DF6278,1 : 200), GPX4 (Affinity, DF6701,1 : 200), NeuN (MilliporeSigma, MAB377X, 1 : 200), 8-hydroxyguanosine (8-OHdG) (Bioss, bs-1278R, 1 : 100), and SIRT1 (Santa Cruz, sc-74465, 1 : 50) in Universal Antibody Diluent (New Cell & Molecular, Suzhou, China) at 4°C overnight. Then, the samples were slowly washed three times with phosphate-buffered saline with 0.5% Tween-20 (PBST) and incubated with corresponding secondary antibodies (Cy™3-conjugated goat antirabbit IgG or goat antimouse IgG, 1 : 200) for 1 h at room temperature (RT). After washing 3 times with PBST, the samples were counterstained with 4,6-diamidino-2-phenylindole (DAPI, MilliporeSigma, 1 : 2000) for 10 min at RT. Fluorescence was visualized by a fluorescence microscope (ZEISS, Scope A1, Germany).

### 2.9. Western Blot Analysis

The total protein samples were extracted from brain tissues of C57BL/6 mice or HT-22 cells for immunoblotting analysis. The samples were lysed in radioimmunoprecipitation assay buffer (RIPA, Beyotime, Nantong, China) containing 1% phenylmethanesulfonyl fluoride (PMSF, Beyotime, Nantong, China) and 1% phosphatase inhibitor cocktails (Sigma-Aldrich, St. Louis, MO, USA). The homogenates after ultrasonic lysis were centrifuged at 15,000 g for 15 min, and the supernatants were collected. Total protein concentrations were measured with the BCA Protein Assay Kit (Beyotime, Nantong, China). An equal amount of protein from each sample was separated by 10% or 12.5% sodium dodecyl sulfate- (SDS-) polyacrylamide gel electrophoresis (PAGE) (EpiZyme, Shanghai, China) and transferred to a 0.22 *μ*m or 0.45 *μ*m polyvinylidene difluoride (PVDF) membrane (MilliporeSigma, Burlington, MA, USA). The membranes were blocked with 5% defatted milk at RT for 2 h and then incubated with primary antibodies against ACSL4 (Proteintech, 22401-1-ap, 1 : 1000), DMT1 (Alpha Diagnostic International, NRAMP24-A, 1 : 1000), TFR (Abcam, ab84036, 1 : 1000), FPN (Novus, NBP1-21502, 1 : 1000), XCT (Proteintech, 26864-1-ap, 1 : 1000), GPX4 (Abcam, ab125066, 1 : 1000), FSP1 (Proteintech, 20886-1-AP, 1 : 1000), CoQ10B (Abcam, ab41997, 1 : 1000), *β*-actin (Bioworld Technology, AP0060, 1 : 5000), and GAPDH (Bioworld Technology, P04406, 1 : 5000) in Universal Antibody Diluent (New Cell & Molecular, Suzhou, China) at 4°C overnight. After the membranes were washed thrice for 30 min in Tris-buffered saline containing Tween 20 (TBST), the membranes were incubated in the appropriate HRP-conjugated secondary antibody (HuaAn Technology, HA1001, 1 : 10000) in Universal Antibody Diluent for 2 h at RT. The protein bands were visualized by western blot detection reagents (MilliporeSigma, Burlington, MA, USA). The ImageJ software quantified band intensities.

### 2.10. Transmission Electron Microscope (TEM)

TEM analysis was performed according to a former report [36]. Briefly, the prepared HT-22 cells were immersed in the TEM-specific fixed solution (2.5% glutaraldehyde). After dehydrating, embedding, and curing, the samples were cut into 70 nm sections. The sections were stained with 4% uranyl acetate for 20 min and 0.5% lead citrate for 5 min. Finally, all sections were observed and photographed with a TEM (Carl Zeiss Thornwood, NY, USA).

### 2.11. Measurement of Malondialdehyde (MDA)

Mice were anesthetized and were transcranial perfused with 50 mL of cold NS. The temporal cortex was collected for assessment. The HT-22 cells were taken for use. Lipid peroxidation was detected by measuring MDA level using an MDA assay kit (Beyotime, S0131M) according to the manufacturer's instructions. An MDA standard curve quantified the levels of MDA. A BCA assay kit measured the protein concentrations.

### 2.12. Measurement of Iron Content

Mice were anesthetized and were transcranial perfused with 50 mL of cold NS. The temporal cortex was taken for use. The brain samples were collected and homogenized with iron assay buffer. The homogenates were centrifuged at 16,000 g for 10 min at 4°C, and the supernatants were collected. The iron content was measured using an iron assay kit (Sigma-Aldrich, MAK025) according to the manufacturer's instructions. The absorbance was measured at 593 nm by spectrophotometry. The iron levels in the samples were determined using a standard curve.

### 2.13. Iron Staining

Iron deposition in HT-22 cells was detected by FerroOrange staining (dojingo, F374). The prepared cells were washed three times with Hanks' Balanced Salt Solution (HBSS) and then stained with 1 *μ*M FerroOrange for 30 min at 37°C. After staining, the samples were rewashed three times with HBSS. Then, the samples were imaged immediately by a fluorescence microscope.

### 2.14. Statistical Analysis

Data were presented as the mean ± standard error of the mean (SEM). Data in each group were analyzed using GraphPad Prism 8.0 statistics software (GraphPad Software Inc., San Diego, CA, USA). Statistical differences in multiple groups were analyzed by one-way analysis of variance (ANOVA) with Tukey's post hoc test. *P* < 0.05 was identified as statistical significance.

## 3. Results

### 3.1. Oxidative Stress-Induced Brain Injury Occurred at an Early Stage following SAH

**Figure 1:**
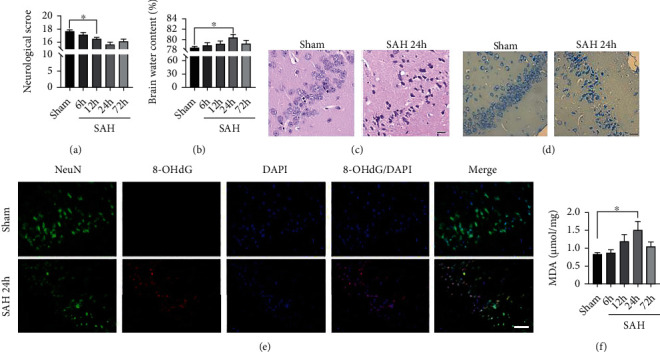
Oxidative damage increased after SAH in the temporal cortex. (a) Quantitative analysis of neurological scores. (b) Quantitative analysis of brain water content. (c, d) Representative images of H&E (c) and Nissl (d) staining at 24 h post-SAH. Scale bars = 20 *μ*m. (e) Representative images of IF staining for 8-OHdG. Scale bars = 50 *μ*m. (f) Quantitative analysis of MDA. *n* = 6/group. Bars represent the mean ± SEM. ^∗^*P* < 0.05 and ^∗∗^*P* < 0.01. H&E: hematoxylin and eosin; MDA: malondialdehyde; 8-OHdG: 8-hydroxyguanosine.

**Figure 2:**
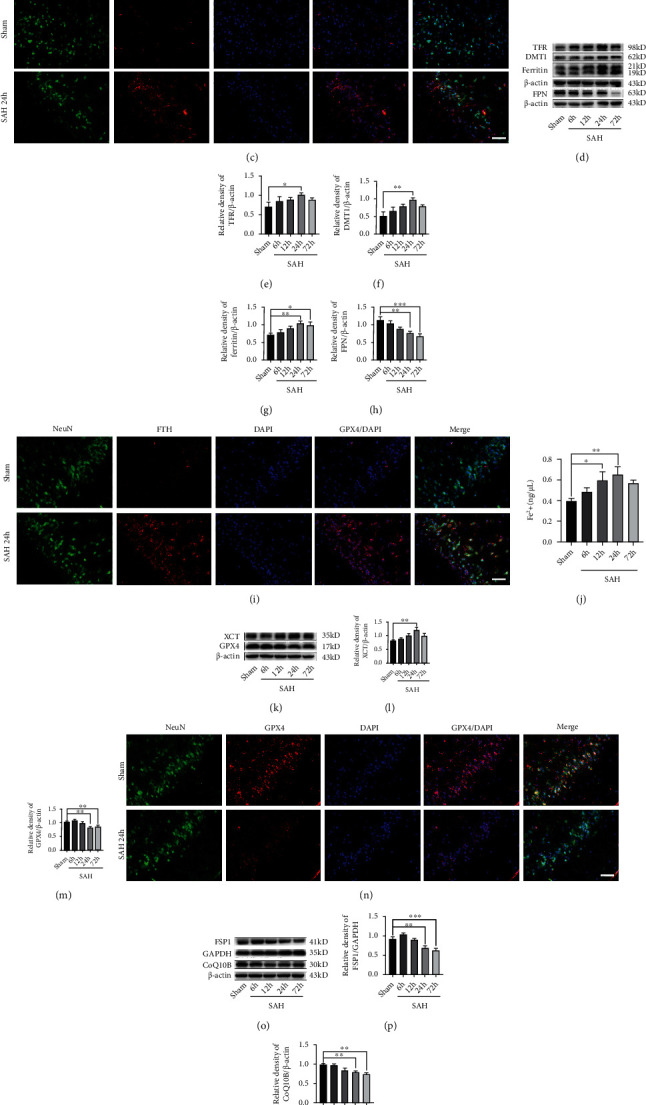
Ferroptosis-related pathways activated in the temporal cortex after SAH. (a) Western blotting assay for ACSL4 expression in the temporal cortex after SAH. (b) Quantification of ACSL4 expression. (c) Representative images of IF staining for ACSL4. (d) Western blotting assay for TFR, DMT1, ferritin, and FPN expression in the temporal cortex after SAH. (e–h) Quantification of the expression of TFR, DMT1, ferritin, and FPN. (i) Representative images of IF staining for FTH. (j) Quantification of ferrous iron content. (k) Western blotting assay for XCT and GPX4 expression. (l, m) Quantification of the expression of XCT and GPX4. (n) Representative images of IF staining for GPX4. (o) Western blotting assay for FSP1 and CoQ10B expression. (p, q) Quantification of the expression of FSP1 and CoQ10B. *n* = 6/group. Bars represent the mean ± SEM. ^∗^*P* < 0.05, ^∗∗^*P* < 0.01, and ^∗∗∗^*P* < 0.001. Scale bars = 50 *μ*m. ASCL4: acyl-CoA synthetase long-chain family member 4; TFR: transferrin receptor; DMT1: divalent metal transporter 1; FPN: ferroportin; FTH: ferritin heavy chain; GPX4: glutathione peroxidase 4; FSP1: ferroptosis suppressor protein 1.

After the mice SAH models were performed, we assessed SAH severity by an 18-point scoring system. The result showed that the neurological score was significantly decreased at 12 h and 24 h post-SAH (*P* < 0.05, compared with the sham group; Figure 1(a)). Moreover, brain edema was the most severe at 24 h after SAH (*P* < 0.05, compared with the sham group; Figure 2(b)). H&E staining also revealed that the morphology of cortical cells in the SAH 24 h group was obviously changed compared with that in the sham group (Figure 1(c)). Then, we performed Nissl staining to evaluate the loss or survival of cortical neurons. We found the neurons with large cellular bodies, and pale nuclei were observed in the sham group, while many damaged neurons with shrunken cellular bodies and dark staining were observed in the temporal cortex at 24 h after SAH (Figure 1(d)). IF staining also showed SAH aggravated oxidative DNA damage in the cortical neurons (Figure 1(e)). Additionally, an increased lipid peroxidation level also exacerbated brain injury after SAH. Indeed, the level of MDA in the SAH 24 h group was significantly higher than that in the sham group (*P* < 0.01; Figure 1(f)), indicating that large amounts of lipid peroxidation accumulated and aggravated oxidative damage at an early stage after SAH.

### 3.2. Ferroptosis-Related Pathways Activated in EBI after SAH

To determine the activation of ferroptosis-related signaling pathways in the brain after SAH, we collected brain tissue samples at different time points after SAH and analyzed the expression level of those proteins by western blotting. Firstly, ACSL4 is an important enzyme that triggers ferroptosis by producing oxidized phosphatidylethanolamines [37]. Moreover, ACSL4 has been identified as a predictor of the cellular initiation of ferroptosis [38]. We found that the expression of ACSL4 level was significantly increased at 24 h after SAH (*P* < 0.05, compared with the sham group; Figures 2(a) and 2(b)). IF staining showed that ACSL4 colocalized with the neuronal marker NeuN in the brain and was significantly increased at 24 h post-SAH (Figure 2(c)).

Iron accumulation, caused by iron metabolism disturbances, is another important mechanism for promoting ferroptosis. Then, we evaluated the process of iron metabolism in the brain after SAH. Western blotting was performed to assess the expression of TFR, DMT1, ferritin, and FPN after SAH in different time-course experiments. As shown in Figures 2(d)–2(h), TFR and DMT1, facilitating iron uptake, were significantly upregulated at 24 h post-SAH (all, *P* < 0.05, compared with the sham group). Besides, FPN also plays a vital role in iron metabolism, which is responsible for the export of iron. We found the FPN level was dramatically decreased at 24 h after SAH compared with that in the sham group (*P* < 0.01). The expression level of ferritin was also significantly increased at 24 h after SAH (*P* < 0.01, compared with the sham group). IF staining showed that the ferritin heavy chain- (FTH-) positive cortical neurons significantly increased after SAH (Figure 2(i)), suggesting iron accumulated in the neurons after SAH. An abnormal increase of intracellular ferrous iron boosted ROS generation in the Fenton reaction, which aggravated ferroptosis [39]. Thus, we also assessed the ferrous iron level in the brain after SAH. We found the ferrous iron level was significantly increased at 12 h post-SAH (*P* < 0.05, compared with the sham group; Figure 2(j)).

Additionally, the inactivation of the cellular antioxidant system is the most important mechanism of ferroptosis. GPX4 is a well-recognized ferroptosis gatekeeper and is crucial in reducing lipid peroxidation. To explore the change of the GPX4 expression after SAH, we also detected the protein level in the brain at different time points following SAH by western blotting. The result showed that the GPX4 level was lowest at 24 h post-SAH (*P* < 0.01, compared with the sham group; Figures 2(k) and 2(m)). However, the expression of XCT at 24 h after SAH was significantly higher than that in the sham group (*P* < 0.01; Figures 2(k) and 2(l)). IF staining also revealed that the GPX4 expressed in the cortical neurons at 24 h after SAH was dramatically decreased compared with that in the sham group (Figure 2(n)).

The FSP1/CoQ10 antioxidative system is a new antiferroptosis mechanism parallel to the GPX4-dependent pathway. Similarly, we also evaluated the activation of FSP1/CoQ10 pathways after SAH by western blotting. As shown in Figures 2(o)–2(q), we found the expressions of FSP1 and CoQ10B levels at 24 h following SAH were significantly lower than those in the sham group (all, *P* < 0.01).

### 3.3. Ferroptosis Pathways Activated in HT-22 Cells following the Stimulation by oxyHb

**Figure 3:**
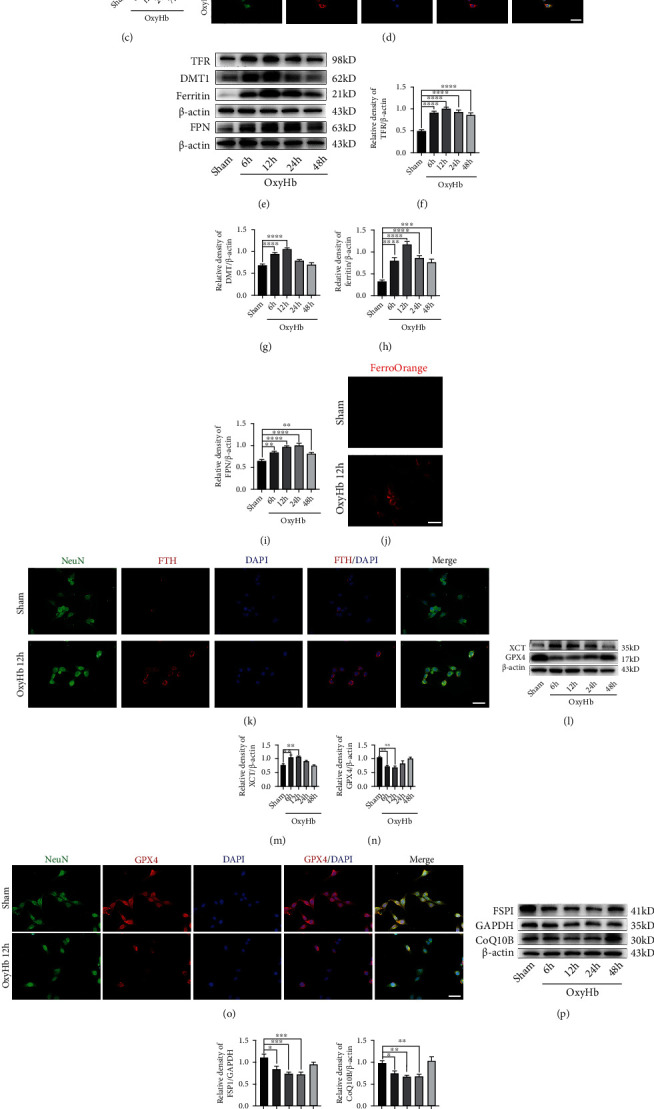
Ferroptosis-related pathways activated in HT-22 cells exposed to oxyHb. (a) Representative images of TEM. (b) Western blotting assay for ACSL4 expression in all groups. (c) Quantification of the expression of ACSL4. (d) Representative images of IF staining for ACSL4. (e) Western blotting assay for TFR, DMT1, ferritin, and FPN expression. (f–i) Quantification of TFR, DMT1, ferritin, and FPN expression. (j) Representative images of ferrous iron staining. (k) Representative images of IF staining for FTH. (l) Western blotting assay for XCT and GPX4 expression in all groups. (m, n) Quantification of XCT and GPX4 expression. (o) Representative images of IF staining for GPX4. (p) Western blotting assay for FSP1 and CoQ10B expression in all groups. (q, r) Quantification of the expression of FSP1 and CoQ10B. (s) Quantitative analysis of MDA. *n* = 6/group. Bars represent the mean ± SEM. ^∗^*P* < 0.05, ^∗∗^*P* < 0.01, ^∗∗∗^*P* < 0.001, and ^∗∗∗∗^*P* < 0.0001. Scale bars = 50 *μ*m. TEM: transmission electron microscope; ASCL4: acyl-CoA synthetase long-chain family member 4; TFR: transferrin receptor; DMT1: divalent metal transporter 1; FPN: ferroportin; FTH: ferritin heavy chain; GPX4: glutathione peroxidase 4; FSP1: ferroptosis suppressor protein 1; MDA: malondialdehyde.

To investigate the role of ferroptosis in neurons after SAH, we used hippocampal neuronal HT-22 cells to perform the *in vitro* experiments. These HT-22 cells were stimulated by 20 *μ*M oxyHb, which simulated SAH *in vivo*. Firstly, we examined the ultrastructure of the HT-22 cells exposed to oxyHb by TEM. As shown in Figure 3(a), the shrunken mitochondria with high-density outer membranes were significantly increased in the cytoplasm of the HT-22 cells after the oxyHb stimulation. Then, the western blotting analysis showed that the expression of ASCL4 reached its highest level at 12 h in HT-22 cells after the stimulation by oxyHb (*P* < 0.01, compared with the sham group; Figures 3(b) and 3(c)). IF staining also suggested that ACSL4 was highly expressed in the cytoplasm after the HT-22 cells were stimulated by oxyHb (Figure 3(d)).

Besides, we also explored the process of iron metabolism in HT-22 cells at 6, 12, 24, and 48 h after the stimulation by oxyHb using western blotting. The results showed that the TFR, DMT1, and ferritin levels were significantly increased at 6 h in HT-22 cells exposed to oxyHb, peaking at 12 h (all, *P* < 0.0001, compared with the sham group; Figures 3(e)–3(h)). However, the expression of FPN was significantly upregulated in HT-22 cells following the stimulation by oxyHb (*P* < 0.01, compared with the sham group; Figure 3(i)). Ferrous iron staining revealed that the oxyHb stimulation exacerbated the accumulation of ferrous iron in HT-22 cells (Figure 3(j)). IF staining further showed that FTH was highly expressed in the cytoplasm of the HT-22 cells after the stimulation by oxyHb (Figure 3(k)).

Furthermore, the antiferroptosis pathways were inhibited in HT-22 cells after the stimulation by oxyHb. The western blotting analysis showed that the XCT level was significantly increased at 6 h and 12 h (all, *P* < 0.01, compared with the sham group; Figures 3(l) and 3(m)); the expression of GPX4 was significantly reduced at 6 h and 12 h after the stimulation of oxyHb (all, *P* < 0.01, compared with the sham group; Figures 3(l) and 3(n)). IF staining also revealed that the GPX4 expression was significantly decreased at 12 h in HT-22 cells exposed to oxyHb (Figure 3(o)). In addition, the FSP1 and CoQ10B levels were significantly reduced at 6 h and were lowest at 12 h and 24 h after the stimulation by oxyHb (all, *P* < 0.05, compared with the sham group; Figures 3(p)–3(r)). Finally, MDA testing further showed that lipid peroxidation dramatically increased at 6 h and 12 h after the HT-22 cells were stimulated by oxyHb (all, *P* < 0.05, compared with the sham group; Figure 3(s)).

### 3.4. Inhibition of Ferroptosis by Fer-1 Alleviated Oxidative Stress-Induced Brain Injury

**Figure 4:**
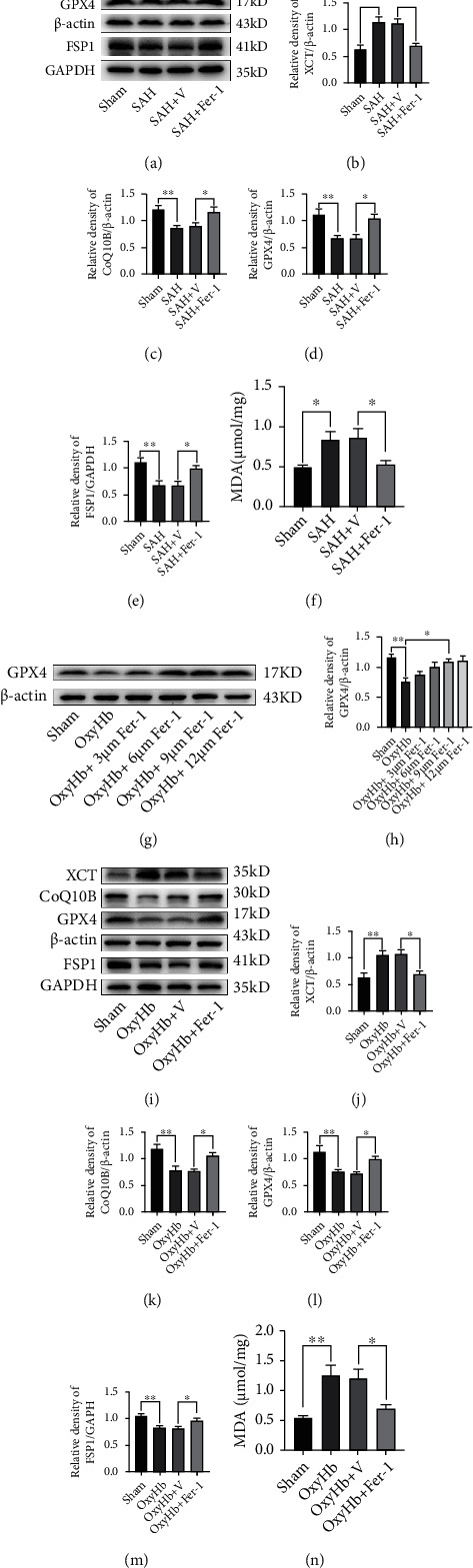
Inhibition of ferroptosis by Fer-1 alleviated oxidative stress-induced brain injury via upregulating the expression of GPX4. (a) Western blotting assay for XCT, GPX4, CoQ10B, and FSP1 expression in different groups *in vivo*. (b–e) Quantification of the expression of XCT, GPX4, CoQ10B, and FSP1. (f) Quantitative analysis of MDA in different groups *in vivo*. (g) Western blotting assay for the expression of GPX4 in all groups with different doses. (h) Quantification of GPX4 expression. (i) Western blotting assay for the expression of XCT, GPX4, CoQ10B, and FSP1 in different groups *in vitro*. (j–m) Quantification of the expression of XCT, GPX4, CoQ10B, and FSP1. (n) Quantitative analysis of MDA. *n* = 6/group. Bars represent the mean ± SEM. ^∗^*P* < 0.05 and ^∗∗^*P* < 0.01. GPX4: glutathione peroxidase 4; FSP1: ferroptosis suppressor protein 1; MDA: malondialdehyde.

The above results showed that ferroptosis mediated neuronal death and was involved in EBI after SAH. We hypothesized that inhibiting the process of ferroptosis could alleviate brain injury after SAH. To verify this hypothesis, we administrated Fer-1, a ferroptosis inhibitor, into the lateral ventricle after establishing the SAH models. The samples from the temporal lobe were collected at 24 h post-SAH. The western blotting analysis indicated the expression of XCT was significantly decreased in the SAH+Fer-1 group (*P* < 0.05, compared with the SAH+V group; Figures 4(a) and 4(b)); the GPX4 level was increased dramatically in the SAH+Fer-1 group (*P* < 0.05, compared with the SAH+V group; Figure 4(d)). In addition, Fer-1 also activated the FSP1/CoQ10 pathway to alleviate brain injury. The bands in the western blotting showed that the expression levels of FSP1 and CoQ10B were significantly increased in the SAH+Fer-1 group (*P* < 0.05, compared with the SAH+V group; Figures 4(c) and 4(e)). The result of MDA also suggested that lipid peroxidation significantly decreased after the administration of Fer-1 (*P* < 0.05, compared with the SAH+V group; Figure 4(f)).

In a dose-response study, Fer-1 was added to the medium containing 20 *μ*M oxyHb at a dosage of 3, 6, 9, and 12 *μ*M. Dose of 9 *μ*M and 12 *μ*M significantly increased the expression level of GPX4 at 12 h after the stimulation by oxyHb (Figures 4(g) and 4(h)). Thus, we used 9 *μ*M Fer-1 for the follow-up *in vitro* experiments because this dose was the lowest dose to provide the maximal effect. As shown in Figures 4(i)–4(m), the results from western blotting showed that the XCT level in the oxyHb+Fer-1 group was significantly lower than that in the oxyHb+V group (*P* < 0.05); the expression levels of GPX4, FSP1, and CoQ10B in the oxyHb+Fer-1 group were considerably higher than those in the oxyHb+V group (all, *P* < 0.05). Moreover, the Fer-1 treatment significantly decreased the production of lipid peroxidation (*P* < 0.05, compared with the oxyHb+V group; Figure 4(n)).

### 3.5. Activation of SIRT1 by RSV Suppressed Ferroptosis following SAH

**Figure 5:**
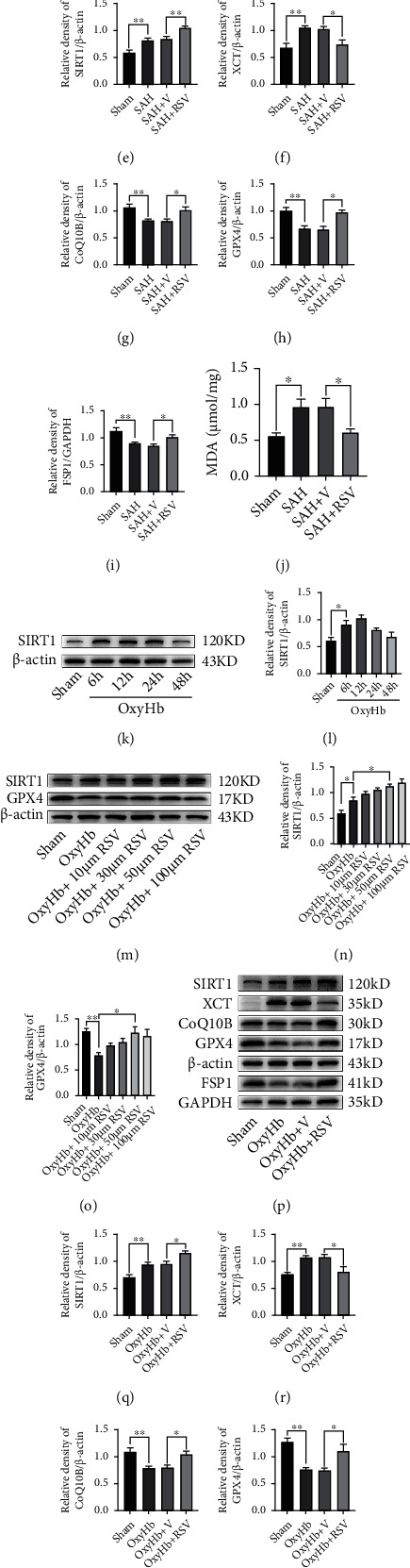
SRIT1 activation by RSV inhibited ferroptosis following SAH *in vivo* and *in vitro*. (a) Western blotting assay for SIRT1 expression in the temporal cortex after SAH. (b) Quantification of SIRT1 expression. (c) Representative images of IF staining for SIRT1. (d) Western blotting assay for SIRT1, XCT, GPX4, CoQ10B, and FSP1 expression in different groups *in vivo*. (e–i) Quantification of SIRT1, XCT, GPX4, CoQ10B, and FSP1 expression. (j) Quantitative analysis of MDA in different groups *in vivo*. (k) Western blotting assay for SIRT1 expression in all groups *in vitro*. (l) Quantification of the expression of SIRT1. (m) Western blotting assay for SIRT1 and GPX4 expression in all groups with different doses. (n, o) Quantification of the expression of SIRT1 and GPX4. (p) Western blotting assay for SIRT1, XCT, GPX4, CoQ10B, and FSP1 expression in different groups *in vitro*. (q–u) Quantification of the expression of SIRT1, XCT, GPX4, CoQ10B, and FSP1. (v) Quantitative analysis of MDA. *n* = 6/group. Bars represent the mean ± SEM. Scale bars = 50 *μ*m. ^∗^*P* < 0.05 and ^∗∗^*P* < 0.01. RSV: resveratrol; GPX4: glutathione peroxidase 4; FSP1: ferroptosis suppressor protein 1; MDA: malondialdehyde.

Previous studies have demonstrated that SIRT1 plays an essential role in the antioxidative response [28, 40, 41]. To explore the time-course change of the SIRT1 expression after SAH, we assessed the SIRT1 level at 6, 12, 24, and 72 h after SAH by western blotting. As shown in Figure 5(a), the expression level of SIRT1 was significantly increased at 24 h post-SAH (*P* < 0.01, compared with the sham group; Figure 5(b)). The expression and distribution of SIRT1 were further identified by IF staining at 24 h following SAH. The result suggested that SIRT1 was weakly expressed in the sham group, while SIRT1 expression was increased in the cortical neurons at 24 h post-SAH (Figure 5(c)). Moreover, SIRT1 was enhanced in the cytoplasm and nucleus after SAH.

Ferroptosis characterizes the lethal accumulation of lipid peroxidation [12]. We assumed that upregulating the expression of the antioxidative protein could reduce the production of lipid peroxidation. Thus, increasing the expression of SIRT1 could reduce lipid peroxidation. We administrated RSV, a SIRT1 activator, into the abdomen of the mice to promote the SIRT1 expression [42]. As shown in Figure 5(d), the results from the western blotting analysis suggested that RSV administration significantly upregulated the SIRT1 expression after SAH (*P* < 0.05, compared with the SAH+V group; Figure 5(e)). Moreover, the expression levels of GPX4, FSP1, and CoQ10B were significantly increased in the SAH+RSV group (all, *P* < 0.05, compared with the SAH+V group; Figures 5(f)–5(i)). MDA analysis also revealed that RSV treatment significantly reduced the production of lipid peroxidation after SAH (*P* < 0.05, compared with the SAH+V group; Figure 5(j)).

In the *in vitro* experiment, we found that SIRT1 was markedly increased at 6 h and 12 h after the exposure to oxyHb (all, *P* < 0.05, compared with the sham group; Figures 5(k) and 5(l)). Similarly, a dose-response study helped determine that 50 *μ*M RSV was the optimal dose in the remaining experiments (Figures 5(m)–5(o)). As shown in Figure 5(p), RSV treatment significantly upregulated the expression of SIRT1 in HT-22 cells (*P* < 0.05, compared with the oxyHb+V group; Figure 5(q)). Furthermore, RSV treatment significantly inhibited ferroptosis in HT-22 cells exposed to oxyHb by increasing the GPX4, FSP1, and CoQ10B levels (all, *P* < 0.05, compared with the oxyHb+V group; Figures 5(r)–5(u)). RSV treatment also significantly decreased the level of lipid peroxidation in HT-22 cells stimulated by oxyHb (*P* < 0.05, compared with the oxyHb+V group; Figure 5(v)).

### 3.6. Inhibition of SIRT1 by SEL Aggravated Ferroptosis following SAH

**Figure 6:**
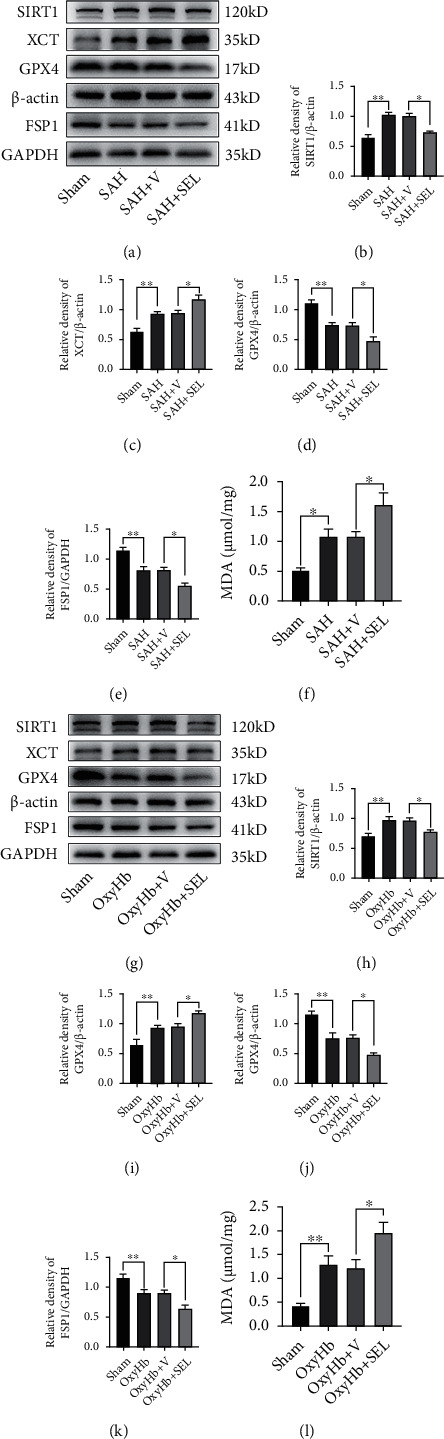
SRIT1 inhibition by SEL aggravated ferroptosis following SAH *in vivo* and *in vitro*. (a) Western blotting assay for SIRT1, XCT, GPX4, and FSP1 expression in different groups *in vivo*. (b–e) Quantification of the expression of SIRT1, XCT, GPX4, and FSP1. (f) Quantitative analysis of MDA in different groups *in vivo*. (g) Western blotting assay for SIRT1, XCT, GPX4, and FSP1 expression in different groups *in vitro*. (h–k) Quantification of the expression of SIRT1, XCT, GPX4, and FSP1. (l) Quantitative analysis of MDA. *n* = 6/group. Bars represent the mean ± SEM. ^∗^*P* < 0.05 and ^∗∗^*P* < 0.01. SEL: selisistat; GPX4: glutathione peroxidase 4; FSP1: ferroptosis suppressor protein 1; MDA: malondialdehyde.

To further determine the antioxidative role of SIRT1 in ferroptosis after SAH, we inhibited the expression of SIRT1 by administrating SEL, a SIRT1-specific inhibitor. Western blotting analysis showed that SEL treatment significantly decreased the SIRT1, GPX4, and FSP1 levels and dramatically increased the expression level of XCT after SAH both *in vivo* and *in vitro* (all, *P* < 0.05; Figures 6(a)–6(e) and 6(g)–6(k)). Moreover, the MDA analysis revealed that the accumulation of lipid peroxidation was significantly increased by SEL treatment both *in vivo* and *in vitro* (all, *P* < 0.05; Figures 6(f) and 6(l)).

## 4. Discussion

Previous clinical and preclinical studies have shown that EBI following SAH is a critical factor affecting the prognosis [30, 43]. Multiple types of neuronal death, including apoptosis, pyroptosis, autophagy, and necrosis, have been determined to be involved in EBI [27, 30]. Ferroptosis is a newly discovered cell death modality that characterizes the accumulation of lipid peroxidation and iron [44]. After SAH, red blood cells released hemoglobin and metabolisms to accelerate the production of superoxide-free radicals and lipid peroxides, leading to severe brain injury [45]. However, the role of ferroptosis in EBI after SAH has not been fully clarified. Although several studies have reported that ferroptosis could exacerbate brain injury after the experimental SAH in the rats, the exact mechanism of ferroptosis has not been studied in the mouse SAH model. In the present study, we established the experimental SAH in the mice by prechiasmatic cistern injection of nonheparinized arterial blood and explored the activation of ferroptosis-related pathways *in vivo* and *in vitro*. Additionally, we also investigated the role of SIRT1 in ferroptosis after SAH. The results of our study suggested that SIRT1 activation could inhibit ferroptosis via upregulating the expression levels of GPX4 and FSP1 after SAH.

ACSL4 is a critical enzyme involved in lipid metabolism to induce ferroptosis by catalyzing the esterification of arachidonoyl or adrenoyl into phosphatidylethanolamines, which generates lipid hydroperoxides [46]. Recent studies have shown that ASCL4-mediated ferroptosis is involved in the pathogenesis of acute brain injuries, including stroke and traumatic brain injury [14, 20, 47–49]. In the controlled cortical impact (CCI) mouse model, the expression of ACSL4 was significantly upregulated at 6 h after injury and mediated neuronal ferroptosis following traumatic brain injury [47]. In the transient cerebral ischemia model, the expression of ACSL4 was suppressed at 1 h and 3 h after ischemia [20], while the ACSL4 level was significantly increased at 24 h after reoxygenation [20, 48]. Moreover, ACSL4 silence was refractory to ferroptosis, protecting against ischemic brain injury. In the present study, we found the ACSL4 level was significantly increased at 24 h post-SAH *in vivo* and at 12 h in HT-22 cells exposed to oxyHb *in vitro*, which was in accordance with the study performed by Qu et al. [14]. Genetic or pharmacological inactivation of ACSL4 inhibited ferroptosis and alleviated neurological deficits after SAH [14, 50]. Thus, ACSL4 may be a potential therapeutic target for ferroptosis inhibition in acute brain injuries.

Iron accumulation, caused by iron metabolism disturbances, is one of the characteristics of ferroptosis. The process of cellular iron regulation is complicated and involves a series of proteins, such as TFR, DMT1, ferritin, and FPN, that collectively modulate the import, neutralization, storage, and export of iron [51]. An abnormal increase of intracellular ferrous iron due to iron metabolism disturbances boosted ROS generation in the Fenton reaction, which triggers the process of ferroptosis [39]. Increasing evidence has demonstrated that iron metabolism disturbances and iron accumulation contribute to the pathogenesis of various brain diseases [52, 53]. Although iron metabolism disturbances and iron overload following SAH have been reported in several studies [39, 54], the present study was the first comprehensive study of iron metabolism after SAH *in vivo* and *in vitro*. We found that TFR, DMT1, and ferritin levels were significantly increased at 24 h post-SAH and at 6 h in HT-22 cells exposed to oxyHb. In a rat SAH model, Li et al. also reported that the expression of TFR was dramatically upregulated at 24 h post-SAH [39]. However, another study showed that the protein level of DMT1 was significantly increased at 48 h after SAH in the rats [54]. The above results indicated that SAH induced the upregulation of iron-uptake proteins, promoting intracellular iron overload. Interestingly, our study showed that the FPN level was significantly decreased at 24 h after SAH *in vivo*, while the expression of FPN was significantly increased at 6 h following the stimulation by oxyHb *in vitro*. A similar result was also reported by Zhang et al. [54]. The upregulation of hepcidin may cause a decreased FPN level in the brain after SAH [54]. Although the excessive intracellular iron could increase the FPN level to export iron out of cells, the expression and function of FPN can be suppressed by hepcidin, a hormone that regulates the iron metabolism [39, 55]. A recent study by Li et al. showed that Fer-1 treatment promoted the FPN expression, decreased the iron level, reduced the production of lipid peroxidation, and improved neurological function [39]. Therefore, iron metabolism disturbances and iron accumulation contribute to ferroptotic neuronal death following SAH. Inhibiting the overexpression of the iron-uptake proteins or upregulating the FPN expression could be another method to alleviate ferroptosis-mediated EBI after SAH.

In addition, the inactivation of the cellular antioxidant system is the most important mechanism of ferroptosis, especially the XCT/GPX4 antioxidant system [56]. GPX4 reduces lipid peroxides, organic hydroperoxides, and hydrogen peroxide by expending GSH. Depletion of GSH and GPX4 inactivation would ultimately result in the accumulation of lipid-based ROS [57]. Therefore, low expression of GPX4 or GPX4 inactivation is a critical downstream step in the process of ferroptosis and the marker of ferroptosis [17]. In this study, we explore the role of GPX4 in a prechiasmatic cistern injection mouse model of SAH for the first time. In agreement with the former study [58], we also found that the expression level of GPX4 was significantly downregulated at 24 h after SAH *in vivo* and at 6 h in HT-22 cells stimulated by oxyHb *in vitro*. Moreover, overexpression of GPX4 significantly attenuated the accumulation of lipid peroxidation and inhibited neuronal death after SAH [58]. Our study also showed that Fer-1 suppressed SAH-induced ferroptosis by upregulating the expression level of GPX4, alleviating oxidative stress-mediated brain injury. XCT, a cystine/glutamate antiporter, also plays a vital role in ferroptosis. The production of GSH in the cells was sustained by the function of XCT, which transfer cysteine into the cell [59]. The depletion of GSH caused by XCT inhibition also triggers ferroptosis [44]. However, we found the expression of XCT was significantly upregulated after SAH in our study, which is contrary to another study of experimental SAH performed by Guo et al. [60]. In a CCI model, XCT was highly expressed after injury [47]. Similarly, XCT was also upregulated after cerebral ischemia-reperfusion [61]. Hence, the expression of XCT in acute brain injury-induced ferroptosis remains to be further explored and clarified.

The FSP1/CoQ10 antioxidative system is a new antiferroptosis mechanism parallel to the GPX4-dependent pathway. Ferroptosis suppressed by FSP1 is mediated by converting CoQ10 to ubiquinol [62]. FSP1 expression positively correlates with ferroptosis resistance [18]. However, the role of the FSP/CoQ10 pathway in ferroptosis after SAH has not been reported so far. Our study is the first study to characterize the FSP/CoQ10-mediated ferroptosis in the pathogenesis of EBI following SAH. We found that FSP1 and CoQ10B expression levels were significantly decreased after SAH *in vivo* and *in vitro*, indicating that the FSP1-mediated ferroptosis may be involved in EBI after SAH. Moreover, our study also showed that Fer-1 could upregulate the FSP1 expression to attenuate SAH-induced ferroptosis.

SIRT1 is a class III histone deacetylase that regulates various cellular functions, including oxidative stress, inflammation, energy metabolism, DNA damage repair, and cell death [63, 64]. Increasing evidence suggests that neuroinflammation is closely associated with the pathogenesis of several neuronal diseases [65, 66]. Extensive studies have demonstrated that SIRT1 has a neuroprotective effect on neuroinflammation-related diseases. For example, Hernández-Jiménez et al. reported that SIRT1 reduced cerebral ischemia-induced neuroinflammation and neuronal death by inhibiting the p53 and NF-*κ*B acetylation [67]. In experimental TBI models, activation of SIRT1 alleviated the inflammatory response by inhibiting NOD-like receptor family pyrin domain containing 3 (NLRP3) inflammasome and mitogen-activated protein kinase (MAPK) pathways [68, 69]. Accumulating studies on SAH have indicated that SIRT1 is widely expressed in the brain and has endogenous neuroprotective effects on EBI by modulating oxidative and inflammatory pathways [26, 27, 70, 71]. Activation of SIRT1 could attenuate EBI and improve neurological function, while SIRT1 silence could exacerbate SAH-induced brain injury. Although our study also showed that the expression of SIRT1 was significantly increased after SAH both *in vivo* and *in vitro*, the elevated SIRT1 level was insufficient to resist ferroptosis in EBI after SAH. To date, little is known about the potential neuroprotective effect of SIRT1 on ferroptosis after SAH. Thus, to investigate the neuroprotective role of SIRT1 in ferroptosis and its underlying molecular mechanism, we upregulated the expression level of SIRT1 by pretreating with RSV and inhibited the SIRT1 expression by SEL pretreatment. We found that artificial overexpression of SIRT1 by RSV significantly reduced the level of lipid peroxidations by upregulating the expression of GPX4 and FSP1, dramatically alleviating ferroptosis. Moreover, inhibiting the activation of SIRT1 by SEL significantly decreased the GPX4 and FSP1 levels and aggravated neuronal ferroptosis. The above evidence suggests that SIRT1 exerts a neuroprotective role in ferroptosis by activating the function of the intracellular antioxidative system. A similar result was also reported by Fang et al. that SIRT1 is involved in the protective effect of calorie restriction on contrast-induced nephropathy by upregulating the expression of GPX4 [41]. Hence, GPX4 might be a new downstream substrate of SIRT1. SIRT1 could represent a new therapeutic target for inhibiting ferroptosis after SAH.

## 5. Conclusions

In the present study, we thoroughly explored the ferroptosis-related pathways in a prechiasmatic cistern injection mouse model of SAH for the first time. Our results showed that ferroptosis was involved in the pathogenesis of EBI after SAH through multiple mechanisms: (1) the upregulated expression of ASCL4, (2) iron overload caused by increasing the expression of iron-uptake proteins (e.g., TFR and DMT1) and decreasing the FPN level, (3) a low expression of GPX4, and (4) the inactivation of the FSP1-mediated antioxidative pathway. Fer-1 treatment following SAH significantly reduced the production of lipid peroxidations and alleviated ferroptosis-mediated neuronal death by activating the expression of GPX4. Moreover, artificial overexpression of SIRT1 significantly inhibited SAH-induced ferroptosis and attenuated EBI. The antiferroptosis effect of SIRT1 could be dependent on the activation of the intracellular antioxidant pathways, which are mediated by increasing the expression level of GPX4 and FSP1.

## Data Availability

All the data supporting the results were shown in the paper and can be available from the corresponding authors.
